# Role of GDF-15, YKL-40 and MMP 9 in patients with end-stage kidney disease: focus on sex-specific associations with vascular outcomes and all-cause mortality

**DOI:** 10.1186/s13293-021-00393-0

**Published:** 2021-09-15

**Authors:** Agne Laucyte-Cibulskiene, Liam J. Ward, Thomas Ebert, Giulia Tosti, Claudia Tucci, Leah Hernandez, Alexandra Kautzky-Willer, Maria-Trinidad Herrero, Colleen M. Norris, Louise Pilote, Magnus Söderberg, Torkel B. Brismar, Jonaz Ripsweden, Peter Stenvinkel, Valeria Raparelli, Karolina Kublickiene, The GOING-FWD Consortium

**Affiliations:** 1 Division of Renal Medicine, Department of Clinical Science, Intervention and Technology, Karolinska Institutet, Stockholm, Sweden; 2 Department of Nephrology, Lund University, Skåne University Hospital, Malmö, Sweden; 3 Institute of Internal Medicine, Catholic University of Rome, Fondazione Policlinico Gemelli IRCCS, Rome, Italy; 4 Department of Biomedical, Experimental and Clinical Sciences, University of Florence, Florence, Italy; 5 Division of Endocrinology and Metabolism, Department of Medicine III, Medical University of Vienna, Vienna, Austria; 6 Clinical and Experimental Neuroscience, Institutes for Aging Research and Bio-Health Research of Murcia, School of Medicine, University of Murcia, Murcia, Spain; 7 University of Alberta, Faculty of Nursing, Edmonton, AB Canada; 8 Cardiovascular and Stroke Strategic Clinical Network, Alberta Health Services, Edmonton, AB Canada; 9 Division of Clinical Epidemiology, Research Institute of McGill University Health Centre, McGill University, Montreal, QC Canada; 10 Clinical Pharmacology and Safety Sciences, R&D, AstraZeneca, Göteborg, Sweden; 11 Division of Medical Imaging and Technology, Department of Clinical Science, Intervention and Technology, Karolinska Institutet, Stockholm, Sweden; 12 Department of Radiology, Karolinska University Hospital in Huddinge, Stockholm, Sweden; 13 Department of Translational Medicine, University of Ferrara, Ferrara, Italy; 14 Division of Renal Medicine, Department for Clinical Science, Intervention & Technology, Karolinska University Hospital-Flemingsberg Campus, 14186 Stockholm, Sweden

**Keywords:** Biomarkers, Calcification, Cardiovascular disease, Chronic kidney disease, End stage kidney disease, TMAO, Uraemia

## Abstract

**Background:**

Sex differences are underappreciated in the current understanding of cardiovascular disease (CVD) in association with chronic kidney disease (CKD). A hallmark of CKD is vascular aging that is characterised, amongst others, by; systemic inflammation, microbiota disbalance, oxidative stress, and vascular calcification—features linked to atherosclerosis/arteriosclerosis development. Thus, it is the necessary to introduce novel biomarkers related to athero-/arteriosclerotic damage for better assessment of vascular ageing in patients CKD. However, little is known about the relationship between uraemia and novel CVD biomarkers, such as growth differentiation factor-15 (GDF-15), cartilage glycoprotein-39 (YKL-40) and matrix metalloproteinase-9 (MMP-9). Therefore, we hypothesise that there are sex-specific relationships between GDF-15, YKL-40, MMP-9 levels in end-stage kidney disease (ESKD) patients in relation to gut microbiota, vascular calcification, inflammation, comorbidities, and all-cause mortality.

**Methods:**

ESKD patients, males (*n* = 151) and females (*n* = 79), not receiving renal replacement therapy were selected from two ongoing prospective ESKD cohorts. GDF-15, YKL-40 and MMP9 were analysed using enzyme-linked immunosorbent assay kits. Biomarker levels were analysed in the context of gut microbiota-derived trimethylamine N-oxide (TMAO), vascular calcification, inflammatory response, oxidative stress, comorbidities, and all-cause mortality.

**Results:**

Increased GDF-15 correlated with higher TMAO in females only, and with higher coronary artery calcification and IL-6. In females, diabetes was associated with elevated GDF-15 and MMP-9, whilst males with diabetes only had elevated GDF-15. No associations were found between biomarkers and CVD comorbidity. Deceased males and females had higher GDF-15 concentrations (*p* = 0.01 and *p* < 0.001, respectively), meanwhile only YKL-40 was increased in deceased males (*p* = 0.02).

**Conclusions:**

In conclusion, in males GDF-15 and YKL-40 were related to vascular calcification, inflammation, and oxidative stress, whilst in females GDF-15 was related to TMAO. Increased levels of YKL-40 and GDF-15 in males, and only GDF-15 in females, were associated with all-cause mortality. Our findings suggest that sex-specific associations of novel CVD biomarkers have a potential to affect development of cardiovascular complications in patients with ESKD.

**Supplementary Information:**

The online version contains supplementary material available at 10.1186/s13293-021-00393-0.

## Background

Chronic kidney disease (CKD) is highlighted as a clinical model of early vascular ageing (EVA) that is associated with exaggerated development of cardiovascular complications. Uraemia-induced proatherogenic profile of circulating biomarkers are related to inflammageing and senescence [1, 2], which alongside traditional risk factors, contributes to a specific phenotype of vascular malfunctions resulting in the increased arterial stiffness [3]. Biological sex is an important determinant of circulating levels of various biomarkers and sex-specific pathophysiological mechanisms [4, 5]. These may occur not only for cardiovascular disease (CVD), but also under uraemia when CVD complications emerge prematurely [6]. Uraemia-induced vascular dysfunction can induce a vicious circle whereby deteriorating kidney function will be accompanied by higher incidence of cardiovascular events and mortality [7]. As a consequence of reduced renal clearance, uraemic toxins accumulate in the body causing additional toxicity to arteries and other organs [8]. Thus, it has been suggested that the uraemia-induced EVA with following adverse cardiovascular outcomes, characterised by; systemic inflammageing, endothelial dysfunction, microbiota disbalance, oxidative stress, vascular smooth muscle cell (VSMC) senescence, and calcification, are linked to atherosclerosis/arteriosclerosis development [9].

Gut microbiota plays an important role in the pathogenesis of CVD as risk factors have been shown to induce gut dysbiosis. Gut dysbiosis through inflammation and activation of the gut–blood barrier leads to increased levels of gut microbiota-derived metabolites, such as trimethylamine N-oxide (TMAO), which has been observed in atherosclerotic CVD [10] and diabetes mellitus [11]. TMAO is a uraemic compound normally filtered by the kidneys, but is found to accumulate with dysfunctional renal status [12]. Although the evidence for sex-specific production of TMAO is mostly reported in animal studies [13, 14], differences in males and females may be related to differences in food eating patterns [15]. Sources of TMAO include ingestion from fish/seafood or indirectly through metabolic conversion of choline and carnitine from food, such as red meat and eggs [12].

Recently, growth differentiation factor 15 (GDF-15) and cartilage glycoprotein 39 (YKL-40) have gained attention as possible biomarkers of vascular remodelling, and thus CVD [16, 17]. GDF-15 belongs to the transforming growth factor ß superfamily and is involved in regulating inflammatory and apoptotic pathways [18], linked to cancer [19], acute and chronic CVD [18], pulmonary conditions [20], and CKD [21]. This biomarker plays an important role in vascular calcification and arterial stiffening in the general population [21–23], and increasing evidence suggests that this factor may serve as a potential marker for kidney failure [24]. However, no significant sex-specific differences have been observed so far [25].

YKL-40, a 40-kDa plasma glycoprotein and a member of the “mammalian chitinase-like proteins”, is also related to inflammatory response [26], and like hsCRP, is not disease specific. Since atherosclerosis has an inflammatory component, it is unsurprising that YKL-40 could be used as a biomarker for identifying the early stages of this disease [26]. Additionally, increased YKL-40 levels have been suggested to serve as a marker of renal function and composite renal outcome [27].

As a finalising detail in this puzzle the disbalance in MMPs, which belong to a large family of endopeptidases that remodel the extracellular matrix (ECM), regulate the activity of many important non-ECM molecules contributing to vascular ageing and remodelling. Their proteolytic activity is regulated at transcriptional and post-translational levels, but also at the tissue level by endogenous inhibitors known as tissue inhibitors of metalloproteinases [28]*.* Increased expression and activation of MMP-9 under inflammatory and oxidative stress conditions plays an important role in atherosclerosis, arterial aneurysm formation, plaque instability, and has been associated with clinical manifestations of CKD and CVD [29, 30]. Moreover, as oestradiol modulates MMP (e.g., MMP-9, MMP-2) activity, as assessed in the development of glomerulosclerosis-associated renal injury [31], the effect of MMP-9 could be sex-specific. In animal models, female sex hormones reduced MMP-2 and MMP-9 activity in aortic tissue and protected from experimental abdominal aortic aneurysm formation [32].

Little is known, especially within sexes, about the relationship of the uraemic phenotype and the three biomarkers GDF-15, YKL-40 and MMP9. Although some studies report increasing levels with kidney function deterioration [33, 34] and vascular remodelling [30], there is still a huge knowledge gap with regard to their interplay with athero-/arteriosclerosis. Both athero-/arteriosclerosis affect arteries in CKD and determine outcomes. Since inflammation, oxidative stress, calcification [35], and gut microbiota [36] are related to vascular ageing, and particularly to these biomarkers, it is important to address all these processes.

We hypothesise that there are sex-specific relationships of GDF-15, YKL-40, MMP-9 with vascular outcomes (athero-/arteriosclerosis) and mortality in end-stage kidney disease (ESKD). We aimed to test whether there are sex differences in circulating biomarkers in patients with ESKD, and whether there are sex-specific differences in GDF-15, YKL-40, MMP-9 levels in relationship to: (1) gut microbiota (by TMAO); (2) coronary artery calcification (by CAC score), and arteriosclerosis (by scoring of medial calcification in epigastric arteries); (3) inflammation (by hsCRP, TNF and IL-6); (4) oxidative stress (by 8-hydroxy-2ʹ-deoxyguanosine; 8-OHdG), and (5) comorbidities and all-cause mortality.

## Methods

### Study population

Among a total of ESKD 340 patients (defined as CKD-EPI eGFR value < 15 mL/min/1.73 m^2^), 230 patients not receiving renal replacement therapy (RRT) (haemodialysis or peritoneal dialysis) were selected from two ongoing prospective CKD cohorts from the Division of Renal Medicine, Karolinska University Hospital, Sweden. One cohort included incident CKD patients [37] awaiting dialysis allocation, and the other included living-donor kidney transplantation cohort [38]. One patient who was recruited subsequently in both cohorts was excluded. Samples used in this study were collected at baseline prior to any dialysis and/or transplantation treatment. The complete study population consisted of both males (*n* = 151) and females (*n* = 79).

The Regional Ethical Committee (EPN), Stockholm, Sweden, approved the study protocols, which were performed in accordance with the Declaration of Helsinki. Written informed consent was obtained from all subjects involved in the study.

### Clinical characteristics

Clinical data were recorded at baseline, at first visit, are presented in Table 1 stratified by sex. Clinical data included information on demographics, medications, comorbidities (CVD and diabetes mellitus), smoking history, in addition to subjective global assessment (SGA), alongside albumin and hand-grip strength measurements, for determination of malnutritional status.

**Table 1:** Clinical, laboratory, and imaging characteristics of the end-stage kidney disease (ESKD) study population stratified by sex

ESKD patients	Female (*n* = 79)	Male (*n* = 151)	*p*
Age, years	55 (42–62)	54 (42–65)	0.16
Cardiovascular disease, *n* (%)	16 (20.3)	52 (34.4)	**0.012**
Diabetes mellitus, *n* (%)	14 (17.1)	43 (28.5)	0.06
Body mass index, kg/m^2^	23.8 (21.5–27.7)	24.3 (22.3–27.7)	0.35
Systolic blood pressure, mmHg	139 (129–152)	146 (135–160)	**0.03**
Diastolic blood pressure, mmHg	82 (74–91)	85 (78–94)	0.06
Smoking history, *n (%)*	10 (12.7)	14 (9.3)	0.61
SGA, > 1 *n* (%)	28 (35.4)	46 (30.5)	0.42
Handgrip strength	20 (17–25)	32 (25–39)	**< 0.001**
eGFR, mL/min/1.73m^2^	5.5 (4.4–8.3)	6.3 (5.1–8.3)	0.07
Medications at cohort entry			
ACEi/ARB, *n* (%)	47 (59.5)	120 (79.5)	**0.001**
β-blockers, *n* (%)	45 (57.0)	104 (68.9)	0.07
Ca-blockers, *n* (%)	47 (59.5)	94 (62.3)	0.68
Statins, *n* (%)	26 (32.9)	60 (39.7)	0.31
Biochemicals			
Total cholesterol, mmol/L	4.7 (4.0–5.3)	4.2 (3.5–4.7)	**< 0.001**
High-density lipoprotein, mmol/L	1.5 (1.2–1.8)	1.1 (0.9–1.4)	**< 0.001**
Triglycerides, mmol/L	1.6 (1.1–2.2)	1.5 (1.2–2.0)	0.79
Apolipoprotein A1, g/L	1.4 (1.3–1.6)	1.3 (1.1–1.5)	**< 0.001**
Apolipoprotein B, g/L	0.9 (0.7–1.0)	0.8 (0.7–1.0)	0.14
Lipoprotein(a), mg/L	327 (102–848)	199 (77–563)	0.18
^†^Albumin, g/L	34.0 (4.6)	34.0 (5.0)	0.92
Creatinine, µmol/L	648 (498–817)	757 (612–922)	**0.001**
^†^Haemoglobin, g/L	109 (13)	107 (12)	0.24
HbA1c, mmol/mol	28 (22–34)	30 (25–39)	0.15
Biomarkers of inflammation, oxidative stress, and uraemic dysfunction			
hsCRP, mg/L	2.1 (0.8–6.9)	2.3 (1.0–8.9)	0.65
IL-6, pg/mL	4.0 (2.3–7.7)	5.9 (2.6–9.5)	0.25
TNF, pg/mL	14.8 (10.9–18.3)	15.6 (12.1–19.5)	0.30
8-OHdG, ng/mL	0.3 (0.2–0.6)	0.2 (0.1–0.3)	**0.03**
TMAO, μM	69.0 (37.7–93.9)	72.6 (48.9–108.0)	0.21
Biomarkers of interest			
GDF-15, ng/mL	4.5 (3.6–5.4)	4.5 (3.4–5.6)	1.00
MMP-9, ng/mL	328.7 (208.0–552.1)	275.8 (168.9–546.1)	0.44
YKL-40, ng/mL	120.4 (86.9–173.2)	114.1 (78.9–187.5)	0.90
Vessel physiology			
CAC score, AU	16.5 (0.0–672.0)	68.5 (0.0–1072.0)	0.13
CAC score, positive *n* (%)	25 (59.5), [*n* = 42]	53 (69.7), [*n* = 76]	0.26
Media calcification, *n* (%)	11 (57.9), [*n* = 19]	33 (84.6), [*n* = 39]	**0.03**
Intimal fibrosis, *n* (%)	3 (15.8), [*n* = 19]	14 (35.9), [*n* = 39]	0.11
Follow-up data			
All-cause mortality, *n* (%)	7 (8.9)	21 (13.9)	0.27

Bold signifies statistical significance *p* < 0.05

Continuous data expressed as median ± quartile range (*Q*1–*Q*3), or ^†^Mean ± SD, and statistical comparisons by Mann–Whitney *U* test and Student’s *t*-test, dependent on not-normal distributed and ^†^normal distributed data

Nominal data expressed as frequency (%) and statistical comparison by Chi-squared test

*ACEi/ARB* angiotensin-converting enzyme inhibitors/angiotensin receptor blockers, *AVC* aortic valve calcification, *CAC* coronary artery calcification, *eGFR* estimated glomerular filtration rate, *GDF-15* growth differentiation factor-15, *HbA1c* glycated haemoglobin, *hsCRP* high-sensitive C-reactive protein, *IL-6* interleukin-6, *MMP-9* matrix metalloproteinase-9, *SGA* subjective global assessment, *TMAO* trimethylamine N-oxide, *TNF* tumour necrosis factor, *YKL-40* 40-kDa plasma glycoprotein, *8-OHdG* 8-hydroxy-2ʹ-deoxyguanosine

All patients underwent non-contrast multi-detector cardiac CT (LightSpeed VCT or Revolution CT; GE Healthcare, Milwaukee, WI, USA) scanning with standard ECG-gated protocol, to evaluate coronary artery calcification (CAC) Agatston scores as described previously [39, 40]. Presence of CAC was defined as total CAC score > 0.

Histological assessment of arterial medial calcification was performed by a pathologist in uraemic vascular biopsies obtained from inferior epigastric arteries in living donor kidney recipients, as presented in our previous paper [41].

### Biochemical measurements

Overnight fasting blood samples were collected in the morning, serum was isolated for necessary analyses, and samples were either analysed immediately or frozen at − 70 °C for future analyses. Biochemical assessments of haemoglobin, albumin, creatinine, blood lipids were measured using routine clinical laboratory techniques. Biochemical measurements are presented in Table 1. For eGFR calculation creatinine-based CKD-EPI equation was used [42].

### Biomarkers of vascular remodelling, inflammation, oxidative stress, and uraemic dysfunction

Three CVD biomarkers, GDF-15, YKL-40, and MMP-9 were analysed in serum using enzyme-linked immunosorbent assay (ELISA) kits. Human GDF-15 Quantikine ELISA kit (DGD150; R&D Systems, UK), Human YKL-40 Quantikine ELISA kit (DC3L10; R&D Systems), and Human MMP-9 Quantikine ELISA kit (DMP900; R&D Systems) were performed according to the manufacturer’s instructions, with minor alterations described herein. Serum samples were diluted fourfold for GDF-15, and 101-fold for both YKL-40 and MMP-9 analyses using the specified diluents in the manufacturer instructions. Patient samples were run as singlets to account for the number of samples. Inter-assay coefficients of variance were 14.2%, 13.9%, and 9.9% for GDF-15, YKL-40, and MMP-9 assays, respectively, calculated from low, medium, and high concentration manufacturers quality control standards included on each assay plate. Intra-assay coefficients of variance were 4.8%, 1.6%, and 2.7% for GDF-15, YKL-40, and MMP-9, respectively, calculated from 16 duplicate samples loaded onto one assay plate for each analyte.

Inflammatory and oxidative stress markers, e.g., hsCRP, TNF and IL-6, 8-OHdG, were measured using routine clinical laboratory techniques. TMAO measurements in serum samples were done via mass spectrometry as previously described [43].

### Statistical analyses

Continuous data are expressed as either median ± interquartile range or mean ± standard deviation dependent on data distribution, either not normal or normal distribution, respectively. Categorical data are expressed at frequency with percentage. All statistical analyses were selected in accordance with the data distribution. For comparing continuous data between males and females non-parametric Mann–Whitney *U* test or parametric Student’s *t*-test were selected. Categorical data were compared using Chi-squared test. Correlation analyses were performed by using Spearman correlation for continuous variables. Linear regression analysis for identification of independent variables associated to analysed biomarkers was used. Statistical analyses were carried out using SPSS (v.27.0, IBM, USA) and R-Commander (Rcmdr; v.3.3).

## Results

### Study population description

This study enrolled patients with ESKD (CKD-EPI eGFR based on creatinine < 15 mL/min/1.73 m^2^), including 79 females and 151 males. As expected, CVD and hypertension were more prevalent in males, and likely the reason why they were treated more often with ACEi/ARB medications (Table 1). Meanwhile females had higher high-density lipoprotein (HDL) concentrations in combination with higher apolipoprotein A1 levels and elevated 8-OHdG biomarker (Table 1). The concentrations of GDF-15, MMP-9 and YKL-40 did not differ between males and females (Table 1).

### Observed sex-specific correlations

Correlation analyses (Additional file 1: Tables S1, S2) identified sex-specific associations between analysed biomarkers and other variables. GDF-15 in males correlated with creatinine-based eGFR (− 0.19, *p* = 0.03), haemoglobin (− 0.18, *p* = 0.04), and hand grip strength (− 0.39, *p* < 0.001) (Additional file 1: Table S1). Surprisingly, MMP-9 in females was related only to diastolic blood pressure (− 0.26, *p* = 0.03) (Additional file 1: Table S2). In males MMP-9 was strongly correlated with glycated-haemoglobin (HbA1C) (0.30, *p* = 0.002) and 8-OHdG (− 0.74, *p* < 0.001), and weakly associated with lipoprotein(a) (− 0.19, *p* = 0.03), albumin (− 0.20, *p* = 0.02) and YKL-40 (− 0.19, *p* = 0.02) (Additional file 1: Table S1). As for YKL-40, in females YKL-40 was associated with triglycerides (0.28, *p* = 0.02), albumin (− 0.27, *p* = 0.02) and haemoglobin (− 0.41, *p* < 0.001) (Additional file 1: Table S2), while in males none of these sex-specific relationships were observed.

### Gut microbiota biomarker TMAO interplay with YKL40 in males and with GDF-15 in females

**Fig. 1:**
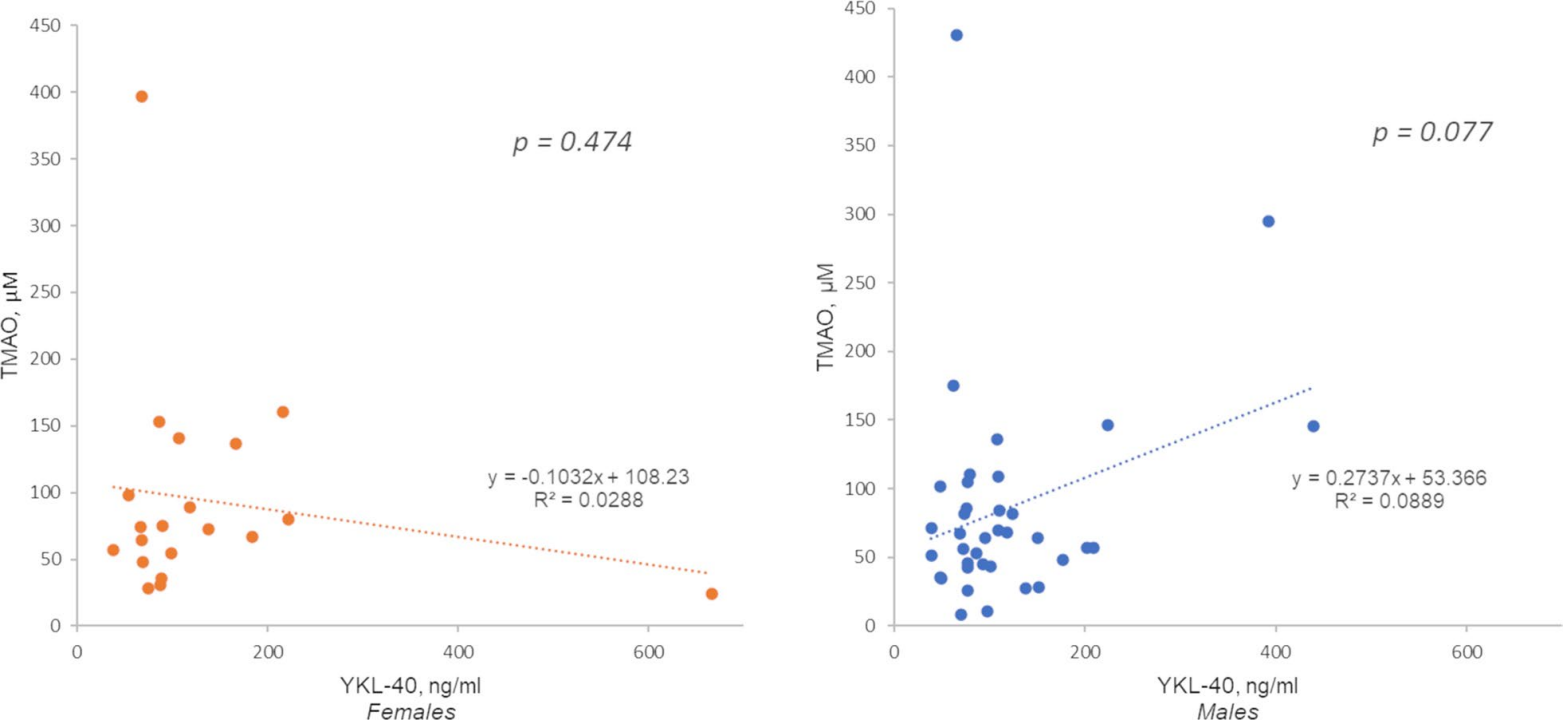
Linear regression analysis with YKL-40 as dependent variable and TMAO—living donor transplantation cohort. Correlation coefficient for males (*n* = 39): *r* = 0.298, *p* = 0.077; for females (*n* = 20): *r* = − 0.169, *p* = 0.474

**Fig. 2:**
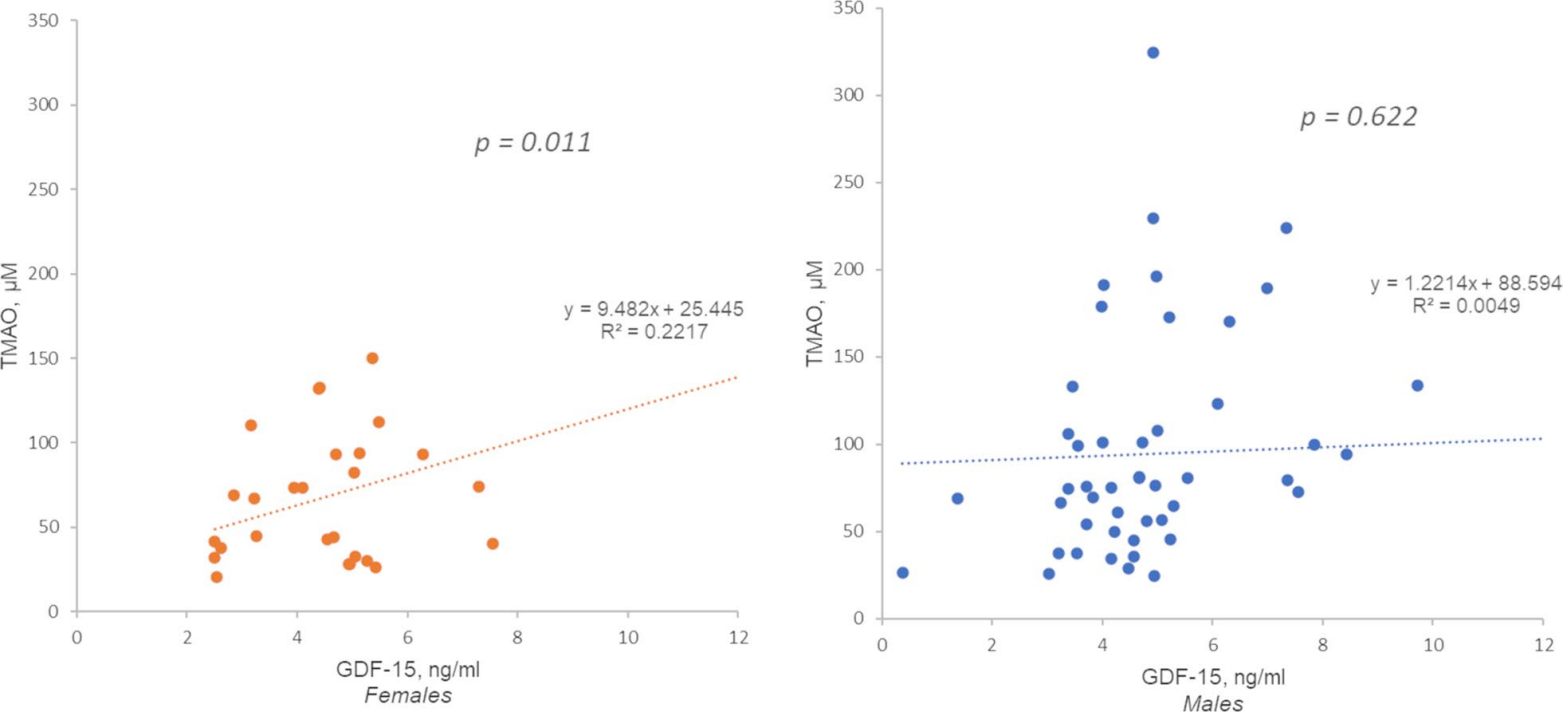
Linear regression analysis with GDF-15 as dependent variable and TMAO. Incident dialysis cohort. Correlation coefficient for males (*n* = 53): *r* = 0.069, *p* = 0.622; for females (*n* = 29): *r* = 0.471, *p* = 0.011

In male patients, from the living-donor transplantation cohort, TMAO was associated with YKL-40 level, although only a trend (*p* = 0.08, Fig. 1.). Conversely, GDF-15 significantly correlated with TMAO in females from the more severe incident CKD cohort (*p* = 0.01, Fig. 2.).

### Sex-specific GDF15 associations with vascular calcification, inflammation, and oxidative stress

**Fig. 3:**
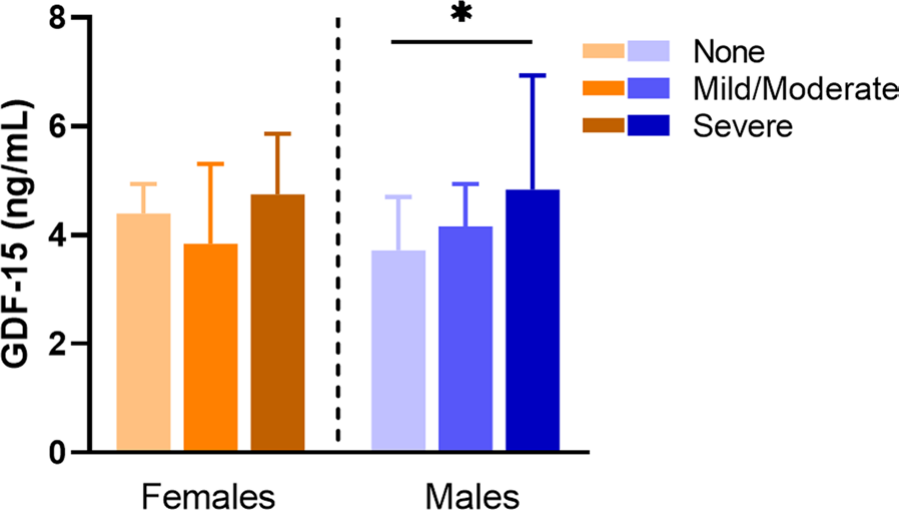
Average GDF-15 concentration in regards of CVD severity in males and females. CVD severity assessed by CT scan and extent of coronary artery calcification set to a nominal scale ranging from none (Agatston score = 0; females *n* = 17; males *n* = 23), mild to moderate (Agatston score = 1–400; females *n* = 13; males *n* = 23), and severe calcification (Agatston score > 400; females *n* = 12; males *n* = 29). Data presented as median (IQR). Kruskal–Wallis; **p < 0.01

Increased GDF-15 levels adjusted to age, kidney function, comorbidities (CVD and DM), and mortality were associated with higher CAC score on CT-scans in males (Table 2). In addition, those males who had CAC score > 400, reflecting severe coronary artery disease, had higher GDF-15 concentration (Fig. 3). We could not find any associations between GDF-15 and arteriosclerosis, determined as medial calcification in epigastric artery biopsies.

**Table 2:** GDF-15 linear regression analysis in males

	Estimate	Standard error	*p*-value
Vascular calcification			
Model 1			
lnCAC score	0.285	0.131	**0.034**
Model 2			
lnCAC score	0.286	0.125	**0.026**
Inflammatory biomarkers			
Model 1			
IL-6	0.199	0.076	**0.011**
hsCRP	− 0.020	0.061	0.747
TNF-alfa	− 0.029	0.044	0.504
Model 2			
IL-6	0.167	0.080	**0.040**
hsCRP	− 0.028	0.061	0.654
TNF-alfa	− 0.019	0.044	0.675

Bold signifies statistical significance *p* < 0.05

Model 1: adjusted for age, cardiovascular disease, diabetes mellitus, kidney function

Model 2: Model 1 + adjusted for mortality

*lnCAC* logarithmic coronary artery calcification, expressed as ln(CAC + 1), *hsCRP* high sensitivity C-reactive protein, *IL-6* interleukin 6, *TNF* tumour necrosis factor

We identified that GDF-15 related with the inflammatory response, reflected by correlations with hsCRP, IL-6 and TNF (Additional file 1: Tables S1, S2). In males, linear regression revealed a pronounced independent IL-6 interplay with GDF-15 adjusted for age, kidney function, comorbidities (CVD, DM) and eventually to all-cause mortality (Table 2). Oxidative stress biomarker 8-OHdG failed to show the same associations.

### GDF-15, MMP-9, and YKL-40 role in comorbidities and nutrition status in females and males

We analysed age-adjusted biomarkers with regard to DM, CVD, and nutrition measures. DM was associated with increased adjusted GDF-15 level in both females and males (females: *ß* = 1.49, SE = 0.60, *p* = 0.02, males: *ß* = 1.34, SE = 0.56, *p* = 0.02), and with MMP-9 specifically in females (*ß* = 208.49, SE = 88.39, *p* = 0.02). CVD was not related to age-adjusted biomarkers.

Since sex-specific relationships of biomarkers and other variables were observed, we performed sex divided linear regression analyses (Table 3). Interestingly, higher age-adjusted MMP-9 activity in males was linked to lower 8-OHdG concentration and lower plasma albumin level and higher HbA1c and remained significant even after adjusting to DM (*p* = 0.02 and *p* = 0.04, respectively, Table 3). Increased YKL-40 concentration in females was associated with lower albumin level, in other words the markers of nutrition and volaemia. GDF-15 was related to albumin level similarly in both males and females.

**Table 3:** Sex divided linear regression models with biomarkers adjusted to age

	Males			Females		
	MMP-9-dependent variable			YKL-40-dependent variable		
	Estimate	SE	*p*	Estimate	SE	*p*
Albumin, g/L	− 17.346	6.520	**0.012**	− 3.852	2.242	0.090
HbA1c, mmol/mmol	6.503	2.841	**0.013**	–	–	–
Haemoglobin, g/L	–	–	–	− 2.794	0.822	**0.001**
8OHdG	− 696.603	213.836	**0.005**	–	–	–

	GDF-15 dependent variable			GDF-15 dependent variable		
	Estimate	SE	*p*	Estimate	SE	*p*
eGFR, mL/min/1.73 m^2^	− 0.184	0.094	0.052	–	–	–
Albumin, g/L	− 0.173	0.048	**0.001**	− 0.106	0.049	**0.035**

Bold signifies statistical significance *p* < 0.05

*eGFR* estimated glomerular filtration rate, *HbA1c* glycated haemoglobin, *8OHgG* 8-hydroxy-2'-deoxyguanosine

### YKL-40 and GDF-15 as sex-dependent biomarkers linked to all-cause mortality in ESKD

**Fig. 4:**
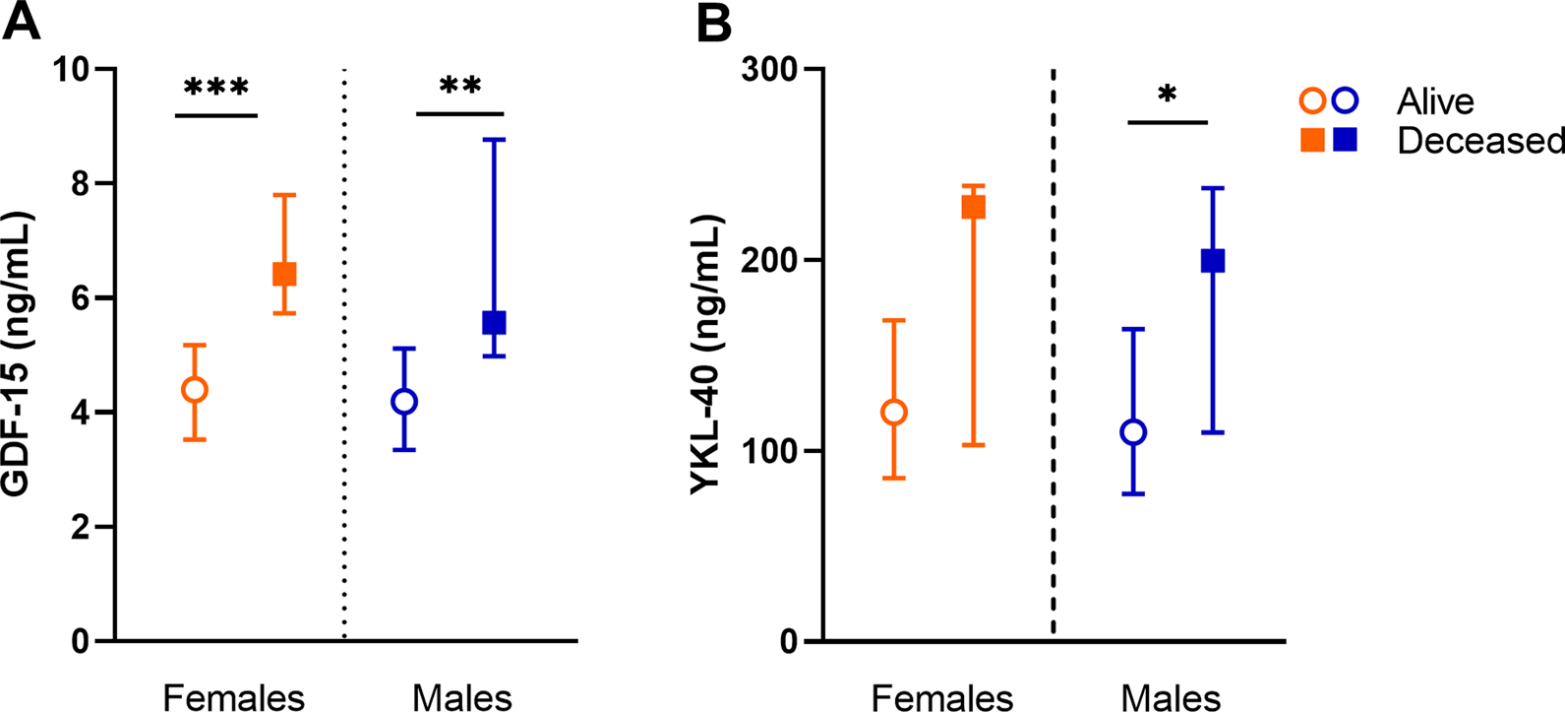
Sex-specific biomarker associations and all-cause mortality. **A** Sex divided GDF-15 level comparison in alive patients (females *n* = 71; males *n* = 127), and deceased patients (females *n* = 7; males *n* = 21). **B** Sex divided YKL-40 level comparison in alive patients (females *n* = 70; males *n* = 123), and deceased patients (females *n* = 7; males *n* = 21). Data presented median (IQR). *p < 0.05, **p < 0.01, ***p < 0.001, age adjusted

All-cause mortality was 21 (13.8%) in males and 7 (8.8%) in females. Higher age-adjusted GDF-15 concentration predicted all-cause mortality in both females and males (*p* = 0.01 and *p* < 0.001, respectively,  Fig. 4A). Elevated YKL-40 level predicted mortality only in males (*p* = 0.02, Fig. 4B).

### Antihypertensive and lipid-lowering treatment effect on GDF15 concentration in females and males

GDF-15 levels were significantly higher in female patients on beta-blockers (*p* = 0.01), calcium channel blockers (*p* = 0.03), or statins (*p* = 0.04) (Additional File 1: Fig. S1A). In males none of the above-mentioned observations were present (Additional File 1: Fig S1B).

## Discussion

The current concept of sexual dimorphism in the uraemic phenotype and its relation to CVD risk needs further clarification. In the present study,  no sex-specific differences of the three novel vascular biomarkers GDF-15, YKL-40, and MMP-9 were observed in patients with ESKD. However, sex-specific associations were found between the analysed biomarkers and specific hallmarks for vascular remodelling, such as vascular calcification, inflammation, oxidative stress, as well as all-cause mortality.

In this study, TMAO was positively associated with higher GDF-15 levels in females. TMAO has been observed in atherosclerotic CVD and in type 2 diabetes mellitus [44]. Thus, it could be speculated that this relationship between TMAO and GDF-15 identifies uraemic females who are undergoing EVA. However, a trend between YKL-40 and TMAO exists in males, stressing the importance of uraemia-induced chronic inflammation and dysbiosis in vascular remodelling in CKD, which can eventually lead to increased cardiovascular risk [45, 46]. Further studies are warranted in larger cohorts to gain further insight to the relationship between TMAO and GDF-15 and/or YKL-40 in CKD patients.

Gut microbiota disintegration is a potential mechanism linked to inflammageing [47], a pro-inflammatory status closely linked to CKD and premature CVD pathology. In our cohort, both GDF-15 and YKL-40 were correlated with one another, age, and inflammatory markers (IL-6 and hsCRP) in both males and females. These biomarkers correlation with inflammation is in concordance with previous studies [26, 48], performed in general population and in CKD patients on dialysis [1, 34], though validation is warranted in a lager cohort. The inflammageing concept also includes oxidative stress, and herein we see a sex-specific association between MMP-9 and 8-OHdG, a nuclear and mitochondrial DNA stress marker, in ESKD males only. The negative relationship might be explained by the compensatory activation of endogenous antioxidants that suppress 8-OHdG [49]. Previously reported within this cohort, which was a sub-group for the current biomarker analysis, 8-OHdG showed a sex-adjusted association with all-cause mortality in CKD patients independent of inflammation markers [50].

The crucial driver of EVA, alongside inflammation and oxidative stress, is the occurrence of vascular calcification [2]. All these drivers show sex-specific associations with GDF-15 levels in ESKD males in the current study. These compelling findings show that GDF-15 levels are associated with greater coronary artery calcification in males only, independent of age, comorbidities, glomerular filtration rate, and mortality. The higher prevalence of CVD among males could explain this relationship followed by both higher GDF-15 and YKL-40 in these patients. Besides, associations between GDF-15 and coronary artery calcium have been reported previously, in both the general population [23] and population without CVD [51], and provide additional prognostic value to cardiac event prediction [52]. Sex disaggregated analyses on GDF-15 are extremely few, however, serum GDF-15 levels have been reported as a predictor of secondary cardiovascular events exclusively in females [53, 54]. This previous investigation, in conjunction with our current study, reports different sex-specific associations of serum GDF-15 levels with CVD incidence in two very different populations (i.e. males with ESKD and females with CVD) [54]. Taken together, the associations of GDF-15 observed here in ESKD males indicate that this may be used as a potential marker of EVA and inflammageing in CKD. Additionally, GDF-15 has shown a positive association with carotid–femoral pulse wave velocity [22], a gold standard for clinical measurement of arterial stiffness that is an early marker of accelerated vascular ageing and subclinical CVD [55]. This highlights that uraemic milieu plays a crucial role in vascular remodelling by completely changing biomarker expression and disease phenotype, and that there is a critical need of further investigations with the essential addition of reporting sex disaggregated analyses.

YLK-40 has previously been described as a predictor of CVD mortality, specifically in DM-type 2 patients [56, 57]. Herein, serum YKL-40 levels displayed sex-specific association in males only with CVD, DM, and all-cause mortality, with only the latter being confirmed in adjusted regressions. YKL-40 is an inflammatory response protein and has been found at elevated levels in patients with CVD [26]. In the aging general population (50–89 years), and aging population with CVD, elevated YKL-40 levels were predictors of all-cause and CVD mortality [58]. Few studies have reported on circulating YKL-40 in the context of CKD, with none reporting on mortality. However, recently a report has shown an association between elevated YKL-40 with the progression of diabetic kidney disease and eGFR decline [57]. To date, no study has explicitly reported sex disaggregated data on the serum levels of YKL-40 in CKD or CVD populations. However, sex differences in plasma YKL-40 levels have been presented in studies assessing neuroinflammation conditions, such as Alzheimer’s disease, where YKL-40 levels were found higher in males [59]. Whilst explicit sex differences in YKL-40 levels alone were not observed in our ESKD cohort, the sex-specific associations explored thereafter could give precedent to study YKL-40 further in a sex disaggregated approach including larger cohorts prospectively based on eGFR through CKD stages.

Surprisingly, no association with MMP-9 and CVD comorbidity was observed in the current study, nor any sex-specific associations with CVD morbidity. Nevertheless, previous studies have observed higher MMP-9 activity in females, compared to males, in various vascular pathologies including advanced coronary atherosclerotic plaques [60] and abdominal aortic aneurysm [61]. MMP-9 activity is also closely related to use of vitamin K antagonists [62], unfortunately data about anticoagulation therapy and/or vitamin K insufficiency status were absent for our study. Further investigation is required to fully assess the role of MMP-9 in CKD, and any sex-specific association with EVA. Tissue inhibitors of MMP-1 should also be included as this marker tightly regulates the activity of MMP-9.

In the current investigation, only patients not receiving renal replacement therapy (haemodialysis or peritoneal dialysis) were selected, because the dialysis has been found to affect the analysed biomarkers. For example, YKL-40 concentration [63] as well as MMP-9 activity [64] decreases after a haemodialysis session. We acknowledge the overall cohort in this study represents a combination of ESKD patients undergoing transplantation, who are appreciated as a lower risk group suitable enough for surgery, meaning their CVD risk burden was relatively lower than those more typical predialysis patient (where a more aggravated vascular phenotype could be expected). Further investigations are warranted to assess the effects of dialysis treatment, both haemodialysis and peritoneal dialysis, and the levels of these novel biomarkers in the contexts of CVD risk burden and sex differences. It is appreciated that TMAO levels can be affected by dietary intake, unfortunately in the current study patients diets were not assessed, future prospective investigations should consider the inclusion of diet assessments when analysing TMAO levels. Finally, with respect to sex comparisons, it should be noted that the reproductive status was not recorded for females included in this study. Therefore, the protective effects of oestrogens that are commonly found at higher levels in pre- and peri-menopausal females, compared to post-menopausal females and males, may play a role in some of the sex differences observed in the current investigation. Future studies should include assessments of reproductive status for females, or better yet, endogenous sex hormone measures for both females and males.

## Perspectives and significance

In summary, we report that in males GDF-15 and YKL-40 were related to vascular calcification and inflammageing, while in females a relationship between GDF-15 and TMAO was observed. Sex-specific associations were observed with higher MMP-9 levels in diabetic females, as well as with higher HbA1c levels in males, together these associations stress a link between long-term hyperglycaemia and EVA. Elevated YKL-40 in males and elevated GDF-15 in both males and females were associated with all-cause mortality. Our data suggest that sex-specific associations exist in relation to GDF-15, YKL-40, and MMP-9, which have the potential to affect the development of vascular complications in ESKD. Further studies are warranted to address sex differences in biomarker levels involved in athero- and arteriosclerosis in different stages of CKD.

## Supplementary Information

**Additional file 1: Table S1.** Male correlation coefficient analysis with applied multiple testing correction. **Table S2.** Female correlation coefficient analysis with applied multiple testing correction. **Fig. S1. **Antihypertensive treatment and statin interplay with mean GDF-15 concentration in females and males. (A) For females, beta-blocker (BB) treatment or not (*n* = 45 and *n* = 34, respectively), calcium-channel blocker (CBB) or not (*n* = 46 and *n* = 32, respectively), and statin treatment or not (*n* = 25 and *n* = 53, respectively). (B) For males, BB treatment or not (*n* = 101 and *n* = 47, respectively), CBB treatment or not (*n* = 91 and *n* = 57, respectively), and statin treatment or not (*n* = 59 and *n* = 89, respectively). Median (IQR), *p < 0.05, **p < 0.01.

## Data Availability

The datasets generated and/or analysed during the current study are available from the corresponding author on reasonable request.
