# Supplementary material for: Role of GDF-15, YKL-40 and MMP 9 in patients with end-stage kidney disease: focus on sex-specific associations with vascular outcomes and all-cause mortality
Source: Biol Sex Differ. 2021 Sep 15;12:50. doi: 10.1186/s13293-021-00393-0 (PMC8444580; doi:10.1186/s13293-021-00393-0)
Supplement: Supplementary file 1 — Additional file 1: Table S1. Male correlation coefficient analysis with applied multiple testing correction. Table S2. Female correlation coefficient analysis with applied multiple testing correction. Fig. S1. Antihypertensive treatment and statin interplay with mean GDF-15 concentration in females and males. (A) For females, beta-blocker (BB) treatment or not (n = 45 and n = 34, respectively), calcium-channel blocker (CBB) or not (n = 46 and n = 32, respectively), and statin treatment or not (n = 25 and n = 53, respectively). (B) For males, BB treatment or not (n = 101 and n = 47, respectively), CBB treatment or not (n = 91 and n = 57, respectively), and statin treatment or not (n = 59 and n = 89, respectively). Median (IQR), *p < 0.05, **p < 0.01. [file 13293_2021_393_MOESM1_ESM.docx]

***ADDITIONAL FILE 1***

**Role of GDF-15, YKL-40 and MMP 9 in patients with end-stage kidney disease: focus on sex-specific associations with vascular outcomes and all-cause mortality.**

Agne Laucyte-Cibulskiene^1,2^*, Liam, J. Ward^1^*, Thomas Ebert^1^, Giulia Tosti^3^, Claudia Tucci^4^, Leah Hernandez^1^, Alexandra Kautzky-Willer^5^, Maria-Trinidad Herrero^6^, Colleen M. Norris^7,8^, Louise Pilote^9^, Magnus Söderberg^1^, Torkel B Brismar^10,11^, Jonaz Ripsweden^10,11^, Peter Stenvinkel^1^, Valeria Raparelli^7,12#^, Karolina Kublickiene^1#†^, on behalf of the GOING-FWD Consortium

* authors contributed equally as first authors.

^#^ authors contributed equally as senior authors.

**List of supplementary material**

**Table S1.** Male correlation coefficient analysis with applied multiple testing correction.

**Table S2.** Female correlation coefficient analysis with applied multiple testing correction.

**Fig. S1.** Antihypertensive treatment and statin interplay with mean GDF-15 concentration in females and males. **(A)** For females, beta-blocker (BB) treatment or not (n = 45 and n = 34, respectively), calcium-channel blocker (CBB) or not (n = 46 and n = 32, respectively), and statin treatment or not (n = 25 and n = 53, respectively). **(B)** For males, BB treatment or not (n = 101 and n = 47, respectively), CBB treatment or not (n = 91 and n = 57, respectively), and statin treatment or not (n = 59 and n = 89, respectively). Median (IQR), * *p* < 0.05, ** *p* < 0.01.

**Table S1.** Male correlation coefficient analysis with applied multiple testing correction.

| **MALES** | **GDF-15 (ng/mL)** | | **MMP-9 (ng/mL)** | | **YKL-40 (ng/mL)** | |
| --- | --- | --- | --- | --- | --- | --- |
|  | **r** | ***P*** | **r** | ***P*** | **r** | ***P*** |
| Age, years | **0.398^**^** | **<0.001** | -0.142 | 0.086 | **0.454^**^** | **<0.001** |
| Body mass index, kg/m^2^ | 0.082 | 0.325 | -0.056 | 0.503 | **0.189^*^** | **0.024** |
| Systolic blood pressure, mmHg | 0.124 | 0.169 | -0.060 | 0.509 | 0.022 | 0.813 |
| Diastolic blood pressure, mmHg | -0.093 | 0.304 | -0.034 | 0.708 | -0.118 | 0.197 |
| Handgrip strength, kg | **-0.385^**^** | **<0.001** | -0.142 | 0.169 | **-0.284^**^** | **0.006** |
| **Measures of vascular calcification** | | | | | | |
| CAC score (AU) | **0.407^**^** | **0.001** | -0.172 | 0.168 | **0.351^**^** | **0.004** |
| Intimal fibrosis score | 0.247 | 0.130 | -0.028 | 0.863 | 0.313 | 0.052 |
| **Laboratory tests** | | | | | | |
| eGFR-creatinine, mL/min/1.73m^2^ | **-0.192^*^** | **0.026** | 0.089 | 0.306 | -0.054 | 0.541 |
| Cholesterol, mmol/L | -0.130 | 0.115 | 0.089 | 0.284 | -0.122 | 0.147 |
| High density lipoprotein, mmol/L | -0.058 | 0.487 | -0.002 | 0.984 | 0.073 | 0.387 |
| Triglycerides, mmol/L | 0.084 | 0.312 | 0.053 | 0.520 | 0.043 | 0.606 |
| Apolipoprotein A1, g/L | -0.112 | 0.177 | 0.018 | 0.824 | 0.045 | 0.589 |
| Apolipoprotein B, g/L | -0.152 | 0.066 | 0.148 | 0.073 | **-0.175^*^** | **0.036** |
| Lipoprotein(a), mg/L | -0.036 | 0.670 | **-0.187^*^** | **0.028** | 0.038 | 0.661 |
| Albumin, g/L | **-0.199^*^** | **0.016** | **-0.202^*^** | **0.015** | -0.118 | 0.159 |
| Creatinine, mmol/L | 0.050 | 0.566 | -0.054 | 0.538 | -0.087 | 0.325 |
| Haemoglobin, g/L | **-0.183^*^** | **0.038** | 0.065 | 0.469 | -0.134 | 0.136 |
| HbA1c, mmol/mol | -0.066 | 0.493 | **0.295^**^** | **0.002** | 0.132 | 0.170 |
| hsCRP, mg/L | **0.318^**^** | **<0.001** | -0.026 | 0.758 | **0.288^**^** | **0.001** |
| IL-6, pg/mL | **0.491^**^** | **<0.001** | -0.101 | 0.291 | **0.360^**^** | **<0.001** |
| TNF, pg/mL | **0.392^**^** | **<0.001** | -0.096 | 0.329 | **0.327^**^** | **0.001** |
| 8-OhdG, ng/mL | 0.103 | 0.665 | **-0.739^**^** | **<0.001** | 0.133 | 0.578 |
| TMAO, μM | 0.173 | 0.108 | 0.072 | 0.512 | 0.147 | 0.183 |
| **Novel biomarkers of vascular remodelling** | | | | | | |
| GDF15, ng/mL | - | - | -0.161 | 0.052 | **0.352^**^** | **<0.001** |
| MMP9, ng/mL | -0.161 | 0.052 | - | - | **-0.194^*^** | **0.022** |
| YKL40, ng/mL | **0.352^**^** | **<0.001** | **-0.194^*^** | **0.022** | - | - |

**Table S2.** Female correlation coefficient analysis with applied multiple testing correction.

| **FEMALES** | **GDF-15 (ng/mL)** | | **MMP-9 (ng/mL)** | | **YKL-40 (ng/mL)** | |
| --- | --- | --- | --- | --- | --- | --- |
|  | **r** | ***P*** | **r** | ***P*** | **r** | ***P*** |
| Age, years | **0.245^*^** | **0.031** | -0.051 | 0.659 | **0.264^*^** | **0.021** |
| Body mass index, kg/m^2^ | -0.028 | 0.808 | 0.041 | 0.720 | 0.139 | 0.228 |
| Systolic blood pressure, mmHg | 0.232 | 0.059 | -0.108 | 0.385 | 0.060 | 0.636 |
| Diastolic blood pressure, mmHg | 0.204 | 0.098 | **-0.263^*^** | **0.032** | -0.038 | 0.763 |
| Handgrip strength, kg | -0.111 | 0.429 | -0.034 | 0.810 | **-0.298^*^** | **0.030** |
| **Measures of vascular calcification** | | | | | | |
| CAC score (AU) | **0.324^*^** | **0.042** | -0.049 | 0.763 | 0.167 | 0.315 |
| Intimal fibrosis score | <0.001 | 1.000 | 0.264 | 0.276 | -0.290 | 0.229 |
| **Laboratory tests** | | | | | | |
| eGFR-creatinine, ml/min/1.73m^2^ | -0.171 | 0.148 | 0.172 | 0.145 | -0.227 | 0.055 |
| Cholesterol, mmol/L | -0.026 | 0.825 | 0.023 | 0.842 | 0.027 | 0.815 |
| High density lipoprotein, mmol/L | -0.015 | 0.899 | 0.054 | 0.639 | -0.127 | 0.275 |
| Triglycerides, mmol/L | 0.127 | 0.272 | 0.029 | 0.804 | **0.275^*^** | **0.016** |
| Apolipoprotein A1, g/L | 0.007 | 0.951 | 0.156 | 0.176 | -0.081 | 0.485 |
| Apolipoprotein B, g/L | -0.036 | 0.755 | -0.118 | 0.307 | -0.011 | 0.924 |
| Lipoprotein(a), mg/L | 0.181 | 0.123 | -0.089 | 0.452 | 0.204 | 0.084 |
| Albumin, g/L | **-0.322^**^** | **0.005** | -0.141 | 0.223 | **-0.267^*^** | **0.021** |
| Creatinine, mmol/L | 0.121 | 0.308 | -0.143 | 0.227 | 0.150 | 0.207 |
| Haemoglobin, g/L | -0.190 | 0.117 | 0.001 | 0.995 | **-0.413^**^** | **<0.001** |
| HbA1c, mmol/mol | 0.058 | 0.654 | 0.108 | 0.403 | -0.173 | 0.174 |
| hsCRP, mg/L | **0.330^**^** | **0.003** | 0.012 | 0.921 | **0.249^*^** | **0.030** |
| IL-6, pg/mL | **0.457^**^** | **<0.001** | 0.032 | 0.801 | **0.410^**^** | **0.001** |
| TNF, pg/mL | 0.236 | 0.070 | -0.015 | 0.910 | **0.300^*^** | **0.022** |
| 8-OhdG, ng/mL | **0.623^*^** | **0.010** | -0.337 | 0.202 | 0.052 | 0.850 |
| TMAO, μM | 0.015 | 0.918 | 0.105 | 0.479 | -0.064 | 0.221 |
| **Novel biomarkers of vascular remodelling** | | | | | | |
| GDF15, ng/mL | - | - | 0.018 | 0.875 | **0.245^*^** | **0.033** |
| MMP9, ng/mL | 0.018 | 0.875 | - | - | 0.142 | 0.221 |
| YKL40, ng/mL | **0.245^*^** | **0.033** | 0.142 | 0.221 | - | - |


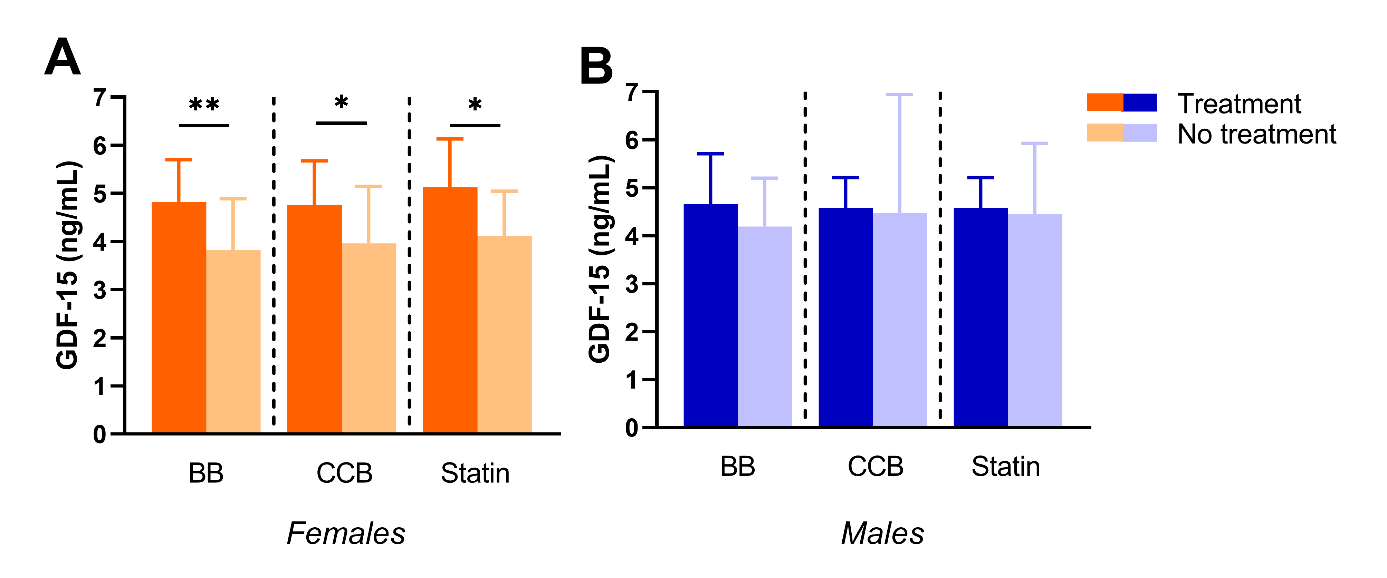


**Fig. S1.** Antihypertensive treatment and statin interplay with mean GDF-15 concentration in females and males. **(A)** For females, beta-blocker (BB) treatment or not (n = 45 and n = 34, respectively), calcium-channel blocker (CBB) or not (n = 46 and n = 32, respectively), and statin treatment or not (n = 25 and n = 53, respectively). **(B)** For males, BB treatment or not (n = 101 and n = 47, respectively), CBB treatment or not (n = 91 and n = 57, respectively), and statin treatment or not (n = 59 and n = 89, respectively). Median (IQR), * *p* < 0.05, ** *p* < 0.01.
